# Enhancing Virulence of an Entomopathogenic Fungus Against the Diamondback Moth, Plutella xylostella (Lepidoptera: Plutellidae), Through Integrated Formulation Strategies Under Laboratory and Greenhouse Conditions

**DOI:** 10.3390/insects17060622

**Published:** 2026-06-12

**Authors:** Muhammad Riaz, Tsui-Ying Chang, Lekhnath Kafle, Wen-Hua Chen

**Affiliations:** 1 Department of Tropical Agriculture and International Cooperation, National Pingtung University of Science and Technology, Pingtung 91201, Taiwan; riazm7419@gmail.com (M.R.); kafle@mail.npust.edu.tw (L.K.); 2 Department of Plant Medicine, National Pingtung University of Science and Technology, Pingtung 91201, Taiwan; whchen@mail.npust.edu.tw

**Keywords:** entomopathogen, diamondback moth, insect mortality, formulation

## Abstract

*Plutella xylostella* (Linnaeus) (Lepidoptera: Plutellidae) is a major cruciferous crop pest worldwide with resistance to multiple insecticide classes, highlighting the need for sustainable alternatives. Entomopathogenic fungi (EPF) are promising biocontrol agents, but their efficacy is limited by slow pathogenicity, environmental sensitivity, and low persistence on insect cuticles. This study evaluated integrated formulation strategies to enhance the virulence of *Beauveria namnaoensis* PM-02 against *P. xylostella* under laboratory and greenhouse conditions. Putative copper and zinc nanoparticle preparations were generated using fungal biomass extracts, with nanoparticle formation inferred from visual changes in the reaction mixtures. Oil-emulsified fungal formulations and combinations with emamectin benzoate were also evaluated. Larval mortality increased significantly with concentration, indicating a clear dose-dependent response. The combined treatment of oil-emulsified fungus and emamectin benzoate, along with emamectin benzoate alone, resulted in the highest larval mortality (100%), whereas fungus alone caused the lowest mortality (43.3%). Lethal concentration (LC_50)_ analysis indicated high toxicity of the combined treatment, while lethal time (LT_50_) values demonstrated more rapid mortality for emamectin benzoate (0.176 days) and the combined treatment (0.830 days) compared with fungus alone (6.25 days). Under greenhouse conditions, the combined treatment showed the highest efficacy, reducing larval survival to 30% and demonstrating enhanced insecticidal activity. Overall, integrated formulation strategies significantly improved fungal efficacy against *P. xylostella*.

## Simple Summary

The diamondback moth is a serious pest of cruciferous crops and is difficult to control because of its rapid life cycle and resistance to chemical insecticides. This study evaluated different strategies to improve the efficacy of the entomopathogenic fungus *Beauveria namnaoensis* PM-02, including nanoparticle formulations, oil-based formulations, and combinations with insecticides. Copper and zinc nanoparticle synthesis was performed using fungal biomass extracts, with putative nanoparticle formation inferred from visual changes in the reaction mixtures. Mortality increased with increasing concentration across all treatments. The highest mortality (100%) was observed in the oil-formulated fungus combined with insecticide treatment, as well as in the insecticide-only treatment, while the lowest mortality (43.3%) occurred in the fungus-only treatment at the lowest concentration under laboratory conditions. Similar trends were observed under greenhouse conditions. Overall, the findings suggest that the biocontrol potential of fungal isolates can be significantly improved through suitable formulation strategies.

## 1. Introduction

*Plutella xylostella* (Linnaeus) (Lepidoptera: Plutellidae) is a globally significant pest of cruciferous crops, characterized by high reproductive potential, rapid generation turnover, and strong dispersal ability [1]. Larval feeding on *Brassica* crops such as cabbage, Chinese cabbage, and cauliflower results in severe defoliation, leaf skeletonization, and substantial yield losses, which can exceed 90% under favorable conditions [2,3]. The economic burden associated with this pest is considerable worldwide, driven by both crop damage and management costs, and is particularly pronounced in regions with continuous cultivation systems, such as Taiwan [4,5].

Chemical insecticides remain the primary tool for managing *P. xylostella* due to their rapid action and effectiveness in reducing immediate crop losses. However, intensive and repeated applications have led to the development of widespread resistance, including cross-resistance to multiple insecticide classes [6,7]. As a result, the long-term sustainability of chemical control strategies has been compromised, highlighting the need for alternative and environmentally sound approaches to pest management.

Entomopathogenic fungi (EPF) are natural pathogens of insects and represent an important component of biological control programs. These fungi exhibit pathogenicity toward a wide spectrum of insect hosts while remaining safe for humans and non-target organisms [8]. Although EPF have demonstrated considerable effectiveness against insect pests, their practical application is often limited by factors such as relatively slow speed of action, sensitivity to environmental conditions (e.g., ultraviolet radiation and humidity), and reduced persistence on the insect cuticle [9]. Consequently, improving the efficacy and virulence of EPF has become a key focus in the development of effective microbial biocontrol strategies.

Emerging nanotechnological approaches offer promising opportunities to improve the performance of microbial agents in pest management. Metal-based nanoparticles, particularly copper and zinc nanoparticles, possess antimicrobial and insecticidal properties and may enhance fungal adhesion, metabolite activity, spore stability, and pathogenicity against insect pests [10,11]. The integration of nanoparticles with entomopathogenic fungi, therefore, represents a promising strategy to improve pest control efficacy while potentially reducing dependence on synthetic insecticides.

In addition to nanotechnology-based approaches, formulation strategies play a critical role in improving the field performance of fungal biopesticides. Oil-based formulations have been shown to enhance the adhesion of fungal conidia to insect cuticles, protect spores from environmental stress, and improve infection efficiency and persistence under both laboratory and field conditions [12]. Furthermore, combining EPF with compatible insecticides at reduced application rates may result in synergistic interactions, thereby improving pest control while reducing reliance on conventional chemical pesticides [13].

Thus, the current study aimed to enhance the pathogenicity of selected entomopathogenic fungi against *P. xylostella* using different formulation strategies. Specifically, the study evaluated the effectiveness of copper- and zinc-based preparations generated through fungal-mediated synthesis, oil-based fungal formulations, and their combinations with selected insecticides. These treatments were compared with conventional pesticide applications and unformulated fungal cultures to determine their relative efficacy and potential for improving EPF-based pest management.

## 2. Materials and Methods

### 2.1. Fungal Isolate

**Figure 1:**
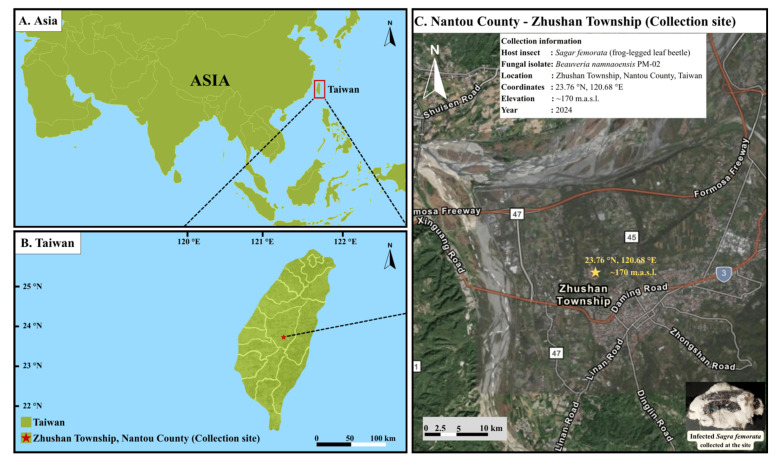
Geographic location of the *Beauveria namnaoensis* PM-02 collection site. The fungal isolate was isolated from an infected *Sagra femorata* (frog-legged leaf beetle) collected in Zhushan Township, Nantou County, Taiwan (23.76 °N, 120.68 °E; ~170 m.a.s.l.) in 2024. The red star in panel B and the yellow star in panel C indicate the collection site.

The fungal strain *Beauveria namnaoensis* PM-02 used in this study was isolated from an infected *Sagra femorata* (frog-legged leaf beetle) collected in Zhushan Township, Nantou County, Taiwan (23.76 °N, 120.68 °E; ~170 m.a.s.l.) in 2024 (Figure 1). The isolate was identified based on morphological characteristics and molecular analysis of the internal transcribed spacer (ITS) region of rDNA, following the procedure described by González-Salgado et al. [14]. Genomic DNA was extracted from one-week-old fungal cultures grown on potato dextrose agar (PDA), and PCR amplification was performed using universal primers ITS1 (5′-TCCGTAGGTGAACCTGCGG-3′) and ITS4 (5′-TCCTCCGCTTATTGATATGC-3′). The obtained sequence was analyzed using the online BLAST tool available through the NCBI database (https://blast.ncbi.nlm.nih.gov/Blast.cgi/ accessed on 1 September 2024) and deposited in GenBank under accession no. PX916452. The isolate was maintained on potato dextrose agar slants and incubated at 28 ± 1 °C under 70–80% relative humidity (RH) for routine culture and preservation.

### 2.2. Synthesis of Copper and Zinc Nanoparticles

Copper (CuNPs) and zinc nanoparticle (ZnNPs) synthesis procedures were performed using the entomopathogenic fungal isolate PM-02. To prepare the fungal biomass extract, *B. namnaoensis* PM-02 was first cultured in potato dextrose broth (PDB) prepared from potato infusion obtained by boiling 200 g of fresh potato in distilled water, followed by supplementation with 20 g glucose per liter. The culture was incubated at 25 ± 2 °C for 7 days under dark conditions. The resulting fungal biomass was collected by filtration, and 50 g of fresh biomass was rinsed twice with sterile distilled water to remove residual culture medium. The washed biomass was then transferred to a 250 mL glass beaker containing 150 mL of sterile distilled water. The suspension was maintained for 5 days to facilitate the release of extracellular metabolites. Following incubation, the mixture was filtered through Whatman No. 1 filter paper to obtain the cell-free fungal filtrate [15]. The cell-free fungal filtrate was subsequently used for the copper- and zinc-based synthesis procedures described below. The final materials evaluated in the bioassays were recovered from reactions between the fungal filtrate and the respective metal salt solutions. These materials likely contained fungal metabolites associated with copper- or zinc-containing particulate matter; however, their exact composition was not determined because detailed physicochemical characterization was not performed.

#### 2.2.1. Copper Nanoparticles (CuNPs)

A fungal-mediated CuNP synthesis procedure was performed following the method by Vivekanandhan et al. [16] with minor modifications. Briefly, 15 mL of fungal culture filtrate was mixed with 85 mL of 1 mM copper sulfate pentahydrate (CuSO_4_·5 H_2_O) solution. The pH of the reaction mixture was adjusted to approximately 8.0 using NaOH and HCl. The mixture was heated at 60 °C for 1 h under continuous magnetic stirring using a Corning PC-620D laboratory stirrer/hot plate (Corning Inc., Corning, NY, USA) and subsequently incubated in the dark at 28 ± 2 °C for 72 h. The color change to dark brown indicated the preliminary formation of nanoparticles. The suspension was centrifuged using an Eppendorf Centrifuge 5810 (Eppendorf AG, Hamburg, Germany) at 11,000 rpm for 8 min, and the resulting pellet was repeatedly washed with double-distilled water to remove impurities. The recovered pellet was air-dried overnight under laminar airflow conditions at 25 ± 2 °C and stored at 4 °C for subsequent use.

#### 2.2.2. Zinc Nanoparticles (ZnNPs)

A zinc nanoparticle synthesis procedure was performed using 10 mL of cell-free fungal filtrate diluted in 100 mL of distilled water. The pH of the solution was adjusted to 9–10 using NaOH and HCl. The culture filtrate was added dropwise to 100 mL of aqueous zinc acetate solution Zn(CH_3_COO)_2_ (1.834 g/100 mL) under constant stirring. The reaction mixture was heated at 100 °C for 6 h with continuous agitation (250 rpm). After synthesis, the suspension was centrifuged at 15,000 rpm for 15 min, and the pellet was collected and repeatedly washed with distilled water. The recovered precipitate was air-dried under laminar airflow conditions at 25 ± 2 °C overnight, finely ground into powder, and stored at 4 °C for subsequent use [17].

#### 2.2.3. Oil-Emulsified Fungal Formulation Preparation

Oil-emulsified fungal formulations were prepared as described by Lei et al. [18] with minor modifications. Conidia were harvested from 10–14-day-old cultures and suspended in sterile distilled water containing 0.01% Tween-80 to obtain uniform conidial dispersions. Conidial concentrations were adjusted to 1 × 10^6^, 1 × 10^7^, and 1 × 10^8^ conidia/mL using a Reichert Bright-Line hemocytometer (Hausser Scientific, Horsham, PA, USA). For preparation of the oil-emulsified formulation, commercial edible vegetable oil (Aishan Enterprise Co., Ltd., Taichung, Taiwan) was added to the conidial suspension at 2% (*v*/*v*), followed by vigorous vortexing to obtain a homogeneous emulsion prior to bioassay application.

### 2.3. Insect Rearing

Eggs of *P. xylostella* were obtained from a laboratory-maintained colony at the Integrated Pest Management Laboratory, Department of Plant Medicine, National Pingtung University of Science and Technology (NPUST), Taiwan. Insect rearing was performed following standard laboratory procedures described previously by Furlong et al. [2] with minor modifications. Egg masses were placed on 6–8-day-old *Brassica* microgreens, and larvae were reared on the same host material until pupation. Pupae were transferred to ventilated containers for adult emergence. Adults were maintained on a 10% honey solution and provided with tissue paper treated with *Brassica* leaf extract to stimulate oviposition. Eggs collected from the treated paper were used for colony maintenance and subsequent experiments. Insects were reared at 25 ± 2 °C, 65 ± 5% relative humidity, and a 12:12 h light:dark photoperiod.

### 2.4. Experiment Treatments

To evaluate the insecticidal efficacy of the selected EPF isolate against *P. xylostella*, multiple formulation strategies were developed, including putatively biosynthesized nanoparticles, oil-emulsified fungal formulations, pesticide-alone treatments using emamectin benzoate, and combined pesticide–fungal oil treatments. Emamectin benzoate was tested at concentrations of 2, 4, and 6 ppm. These concentrations were intentionally selected to evaluate the efficacy of reduced pesticide inputs when used alone or in combination with EPF. Experimental treatments are summarized in Table 1.

### 2.5. Laboratory Bioassay Against P. xylostella

Third-instar larvae of *P. xylostella* were used for all laboratory bioassays following procedures modified from Wakil et al. [13] and Chepkemoi et al. [19]. For fungal-based treatments, including nanoparticles, oil-emulsified, and fresh fungal formulations, larvae were immersed in the respective suspensions for 5 s and then transferred onto fresh cabbage leaves placed in Petri dishes. For pesticide treatments, cabbage leaves were dipped in the respective pesticide solutions and air-dried at room temperature before larval introduction. In combined treatments, cabbage leaves were first treated with pesticide solutions and allowed to air-dry, after which larvae were immersed in the oil-emulsified fungal suspension for 5 s before being transferred onto the treated leaves. Larvae in the control group received only sterilized water treatment. Each treatment consisted of three replicates with 10 larvae per replicate. Surface-sterilized fresh cabbage leaves were provided as food throughout the experiment. All experimental units were maintained at 25 ± 1 °C, 70–80% relative humidity, and a 12:12 h (light:dark) photoperiod. Larval mortality was recorded at 24 h intervals for 7 days [20]. The experiment was independently repeated to ensure reproducibility.

### 2.6. Greenhouse Bioassay

A greenhouse experiment was conducted in March 2026 at the greenhouse facility of NPUST, Taiwan (22°38′48″ N, 120°36′12″ E), to evaluate the efficacy of selected treatments against *P. xylostella* following a procedure modified from Thakur et al. [21]. Based on laboratory bioassay results, four treatments were selected for further evaluation: oil-emulsified EPF (1 × 10^7^ conidia/mL), oil-emulsified EPF combined with pesticide (4 ppm), pesticide alone (4 ppm), and freshly prepared fungal suspension (1 × 10^7^ conidia/mL). Healthy, disease-free cabbage seedlings were transplanted into pots and maintained under greenhouse conditions until the 5–6-leaf vegetative stage. Third-instar larvae of *P. xylostella* were used for the bioassay. The experiment was arranged in a completely randomized design (CRD) with 12 plants per treatment, and each plant served as an experimental replicate. Five larvae were released onto each plant. For pesticide treatments, cabbage leaves were sprayed with pesticide solution and allowed to air-dry before larval introduction. For combined treatments, leaves were first sprayed with pesticide solution and allowed to dry, after which larvae were immersed in the oil-emulsified fungal suspension for 5 s before transfer onto treated plants. Larvae in the control group received only a sterilized water treatment.

**Figure 2:**
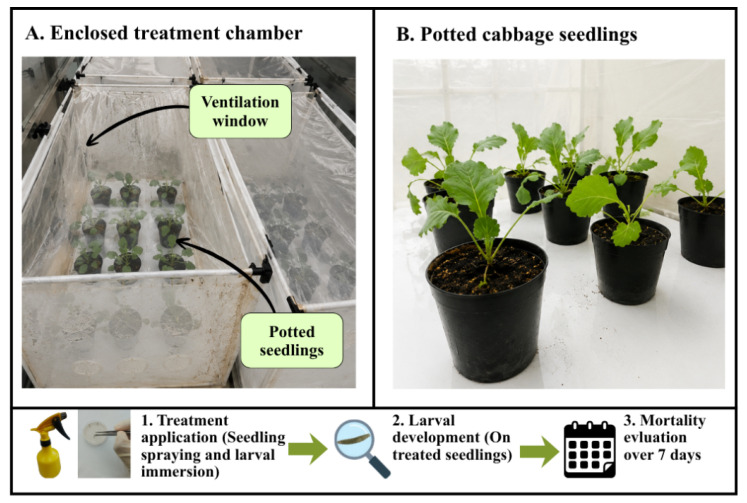
Experimental setup of the greenhouse insect bioassay using third-instar larvae of *Plutella xylostella*: (**A**) enclosed treatment chambers used for insect exposure; (**B**) potted cabbage seedlings used for treatment applications, evaluated over 7 days.

Each treatment was maintained in a separate rearing cage (60 × 60 × 120 cm; mesh aperture 700 µm) to prevent larval escape during the experiment. Following larval inoculation, each plant was covered with a perforated plastic bag (40 × 30 cm) to restrict larval movement while allowing adequate air exchange (Figure 2). Greenhouse temperatures averaged approximately 20 °C at night and 29 °C during the day, with 55–65% RH under natural photoperiod conditions. Plants were irrigated periodically to maintain adequate soil moisture, and no fertilizers were applied during the experimental period. Larval mortality was recorded daily for 7 days post-treatment, and mortality data were corrected using Abbott’s formula [22].

### 2.7. Statistical Analysis

Insect mortality was corrected using Abbott’s formula [22]. Effects of treatment, conidial concentration, and exposure time on larval mortality were analyzed using three-way analysis of variance (ANOVA), followed by Tukey’s HSD test for pairwise comparisons at *p* < 0.05 [19]. The median lethal concentration (LC_50_) and median lethal time (LT_50_) values were estimated through probit analysis in SPSS v23.0 (IBM Corp., Armonk, NY, USA). Kaplan–Meier survival analysis was applied to evaluate temporal mortality patterns, and differences among treatments were tested using log-rank and Wilcoxon (Breslow) tests in GraphPad Prism 9 [23]. Additional statistical analyses were performed using Statistix 8.1 (Analytical Software, Tallahassee, FL, USA).

## 3. Results

### 3.1. Synthesis and Visual Confirmation of Copper and Zinc Nanoparticles

**Figure 3:**
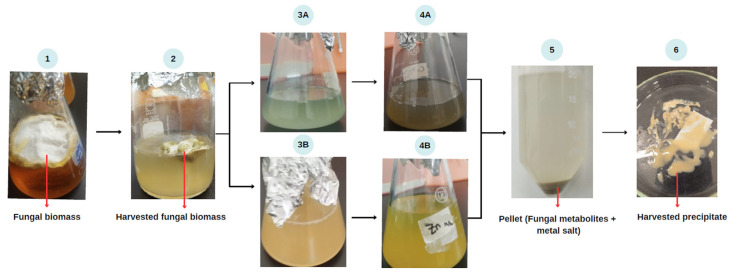
Workflow of fungal-mediated synthesis and recovery of nanoparticles. (**1**,**2**) Preparation of fungal culture filtrate; (**3A**,**3B**) addition of metal precursors (copper sulphate and zinc acetate); (**4A**,**4B**) color change indicating possible nanoparticle formation; (**5**) centrifugation to obtain pellets; (**6**) dried nanoparticle material.

Copper nanoparticle-like suspensions were prepared using fungal culture filtrates following a green synthesis approach of strain PM-02. Preliminary nanoparticle formation was indicated by a color change in the reaction mixture from light blue to dark brown after 72 h of incubation. Similarly, a pale white precipitate was observed during the zinc nanoparticle synthesis process following heat treatment. After centrifugation and repeated washing, the recovered pellets were dried and used for subsequent experiments (Figure 3).

### 3.2. Mortality and Survival Analysis

**Figure 4:**
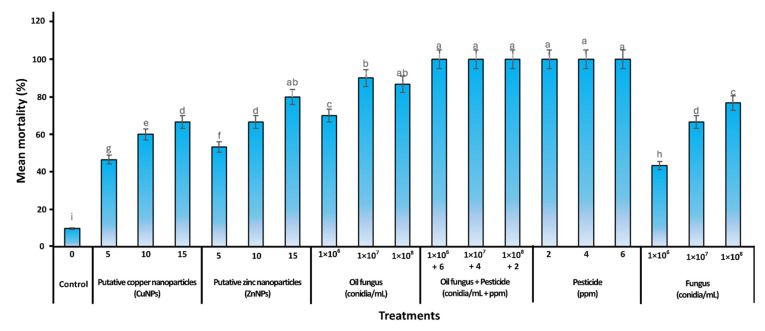
Overall mortality (%) of third-instar larvae of *Plutella xylostella* exposed to different treatments over 7 days. Values represent mean ± standard error (SE). Means followed by the same lowercase letter are not significantly different according to Tukey’s HSD test (*α* = 0.05).

The ANOVA revealed significant differences in larval mortality among treatments and concentration combinations, including the control (F_18,38_ = 13.70, *p* < 0.01). Mortality increased with increasing conidial concentration, indicating a clear dose-dependent response. Maximum mortality (100%) was recorded in the combined oil-formulated fungal and pesticide treatment, as well as in the pesticide-alone treatment, after 7 days of exposure. In contrast, the lowest mortality (43.3%) was observed in larvae treated with the fungal suspension alone at 1 × 10^6^ conidia/mL. Analysis of treatment effects further demonstrated significant differences in larval mortality among treatments (F_5,51_ = 26.50, *p* < 0.001), indicating marked variation in treatment efficacy (Figure 4).

**Figure 5:**
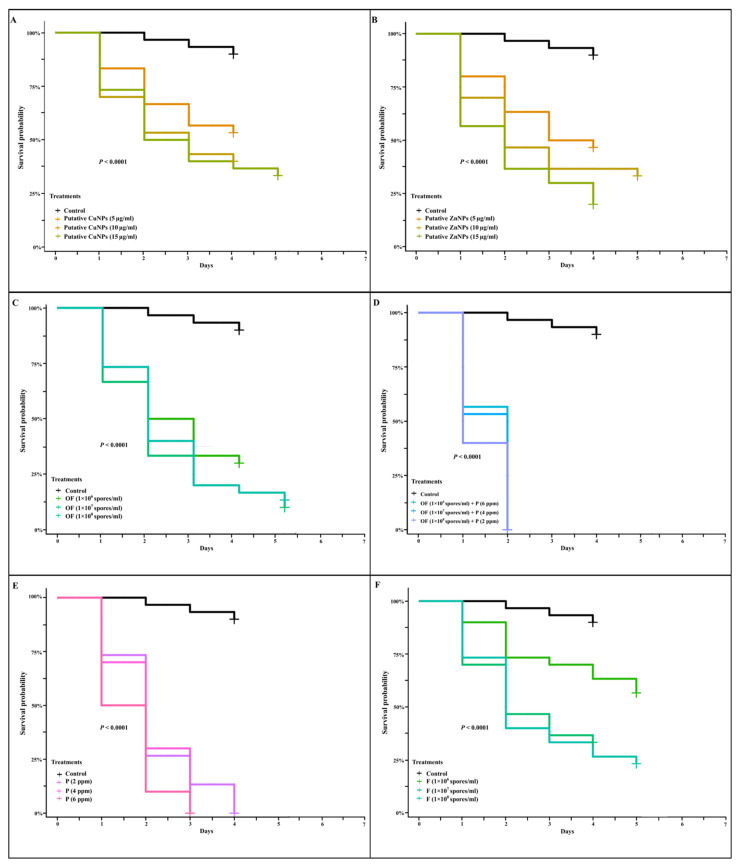
Kaplan–Meier survival curves of third-instar larvae of *Plutella xylostella* following exposure to different treatments: (**A**) Putative copper nanoparticles (CuNPs), (**B**) Putative zinc nanoparticles (ZnNPs), (**C**) Oil-emulsified fungus, (**D**) Emulsified fungus combined with pesticides, (**E**) pesticides alone, and (**F**) Fungus alone, evaluated across multiple concentrations over 7 days.

Kaplan–Meier survival analysis revealed significant differences in larval survival among treatments. Oil-emulsified EPF combined with pesticide (emamectin benzoate), as well as emamectin benzoate alone, caused rapid declines in larval survival, with survival reaching 0% within approximately 2–3 days of exposure. In contrast, larvae treated with the fungal suspension alone at 1 × 10^6^ conidia/mL and putative CuNPs at 5 µg/mL showed the highest survival probabilities (approximately 56% and 53%, respectively), indicating comparatively lower toxicity at these concentrations. The log-rank (Mantel–Cox) test revealed significant differences among concentrations for putative CuNPs (*χ*^2^ = 22.36, df = 3, *p* < 0.01), fungal suspension (*χ*^2^ = 161.92, df = 9, *p* < 0.01), oil-formulated fungus (*χ*^2^ = 47.02, df = 3, *p* < 0.01), oil-formulated fungus combined with pesticide (*χ*^2^ = 86.21, df = 3, *p* < 0.01), pesticide treatment (*χ*^2^ = 90.22, df = 3, *p* < 0.01), and putative ZnNPs (*χ*^2^ = 33.12, df = 3, *p* < 0.01) (Figure 5). Overall, the treatments produced significant dose-dependent reductions in larval survival, with both the combined treatment and pesticide-alone treatment exhibiting the most rapid reductions in survival probability.

### 3.3. LC_50_ and LT_50_ Analysis

Substantial differences in toxicity were observed among treatments. The combined oil-formulated fungus and pesticide treatment produced extremely high mortality across all tested concentration combinations, preventing reliable LC_50_ estimation because of insufficient variation in the dose–response data. Among the treatments for which LC_50_ values could be estimated, pesticide treatment alone showed the greatest toxicity (LC_50_ = 0.088), followed by putative ZnNPs (7.18 µg/mL) and putative CuNPs (9.00 µg/mL). In contrast, fungal treatments alone showed comparatively lower toxicity, with LC_50_ values reaching 6.91 × 10^6^ conidia/mL. All estimable treatments exhibited significant dose–response relationships (*p* < 0.01) (Table 2).

The LT_50_ values differed significantly among treatments, indicating variation in the speed of insecticidal activity. Pesticide treatment alone showed the lowest LT_50_ value (0.176 days; 95% CI: 0.035–0.399), indicating the fastest estimated mortality response according to the fitted probit model. The combined oil-formulated fungus and pesticide treatment also exhibited a relatively low LT_50_ value (0.830 days; 95% CI: 0.48–1.22), indicating rapid insecticidal activity. Overall, substantial differences in mortality dynamics were observed among treatments (Table 3).

### 3.4. Greenhouse Trial

**Figure 6:**
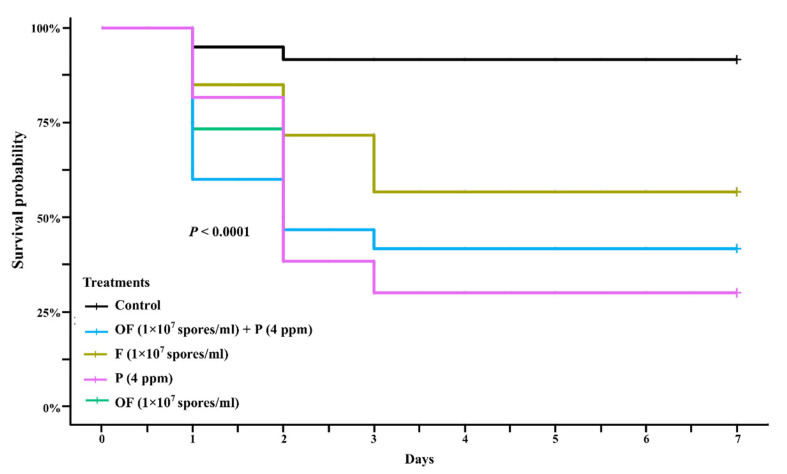
Kaplan–Meier survival curves of third-instar larvae of *Plutella xylostella* exposed to different treatments under greenhouse conditions over 7 days. Treatments included oil-emulsified entomopathogenic fungi (OF), oil-emulsified fungi combined with pesticide (OF + P), fungus alone (F), and pesticide alone (P). The control group was untreated.

Kaplan–Meier survival analysis revealed significant differences in larval survival among treatments over the 7-day observation period under greenhouse conditions. Larval survival declined progressively across all treatment groups, whereas the control maintained the highest survival rate (>90%), indicating minimal natural mortality. Among the treatments, the combined application of fungus (1 × 10^7^ conidia/mL) and pesticide (4 ppm) resulted in the most rapid decline in survival, reaching approximately 30% by day 3. Pesticide treatment alone also caused substantial mortality, reducing survival to approximately 37%. The oil-formulated fungal treatment showed moderate efficacy, with survival remaining at approximately 42%, whereas the fungal treatment alone resulted in comparatively higher survival (approximately 57%) (Figure 6).

**Figure 7:**
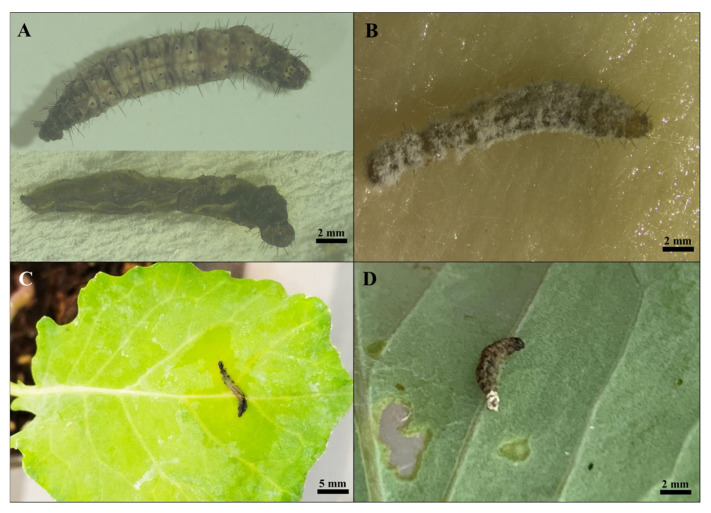
Larval mortality and fungal infection under laboratory and greenhouse conditions. (**A**) Dead larva (laboratory); (**B**) Fungal growth on larva (laboratory); (**C**) Dead larva (greenhouse); (**D**) Fungal growth on larva (greenhouse).

The log-rank (Mantel–Cox) test confirmed significant differences in survival distributions among treatments, including fungus (*χ*^2^ = 18.23, df = 1, *p* < 0.01), oil-formulated fungus (*χ*^2^ = 33.15, df = 1, *p* < 0.01), fungus + pesticide (*χ*^2^ = 34.22, df = 1, *p* < 0.001), and pesticide alone (*χ*^2^ = 45.68, df = 1, *p* < 0.01). Similarly, the Wilcoxon (Breslow) test also showed significant differences among treatments, including fungus (*χ*^2^ = 17.12, df = 1, *p* < 0.01), oil-formulated fungus (*χ*^2^ = 31.28, df = 1, *p* < 0.01), fungus + pesticide (*χ*^2^ = 33.06, df = 1, *p* < 0.01), and pesticide alone (*χ*^2^ = 41.98, df = 1, *p* < 0.01), indicating stronger treatment effects during the early exposure period. Overall, all treatments significantly reduced larval survival, with the combined fungus–pesticide treatment producing the greatest reduction under greenhouse conditions (Figure 6 and Figure 7).

## 4. Discussion

The present study demonstrated that the efficacy of EPF against *P. xylostella* can be significantly improved through formulation strategies, including nanoparticle-based formulations, oil formulations, and combined applications with chemical pesticides. The results further indicated that treatment type, concentration, and formulation collectively influenced larval mortality. Under laboratory bioassay conditions, application of unformulated fungal suspensions produced a clear dose-dependent mortality response, with mortality increasing as conidial concentration increased. The highest concentration (1 × 10^8^ conidia/mL) resulted in the greatest mortality compared with lower concentrations. However, mortality remained moderate (approximately 76.7%) after the exposure period, suggesting that a longer infection period may be required to achieve higher mortality levels. Similar mortality patterns have been reported previously [24,25], which may be associated with the progressive infection process of EPF involving conidial adhesion, cuticle penetration, internal colonization, and subsequent host death.

The role of putative CuNP and ZnNP preparations in influencing insect mortality was also evaluated in this study. Both putative nanoparticle preparations exhibited dose-dependent insecticidal activity, with mortality increasing as concentration increased. However, overall mortality remained moderate across treatments. Among the two putative nanoparticle preparations, the ZnNP treatment showed comparatively higher efficacy, achieving approximately 80% mortality at the highest concentration (15 µg/mL), indicating greater insecticidal potential than putative CuNPs. Nanotechnology has emerged as a promising alternative to conventional chemical pesticides for sustainable pest management, and previous studies have demonstrated the effectiveness of biosynthesized and chemically synthesized nanoparticles against various agricultural insect pests and disease vectors [26]. For instance, Vivekanandhan et al. [16] reported significant larvicidal activity of fungus-mediated CuNPs against mosquito species, including *Anopheles stephensi*, *Aedes aegypti,* and *Culex quinquefasciatus*, as well as against *Tenebrio molitor*. Similarly, Pittarate et al. [27] demonstrated strong larvicidal activity of chemically synthesized ZnNPs against *Spodoptera frugiperda* under laboratory conditions. These findings are consistent with the present results, where putative ZnNPs exhibited relatively greater efficacy than putative CuNPs. Although the physicochemical properties of the materials generated in the present study were not characterized, previous studies have attributed the insecticidal activity of characterized nanoparticles to their small particle size and high surface reactivity, which can disrupt physiological and cellular processes in insects through oxidative stress and cellular damage [10]. Despite their demonstrated toxicity, nanoparticle treatments alone were less effective than combined EPF-pesticide treatments in the present study, suggesting that nanoparticles may be more suitable as supportive components within integrated pest management (IPM) systems rather than as standalone control agents.

Incorporation of oil into the EPF suspension significantly enhanced fungal efficacy, resulting in mortality levels of up to 90% in the present study. This improvement is likely associated with the role of oil as an adjuvant that enhances conidial adhesion to the insect cuticle. Oils possess chitinophilic properties that improve the affinity between hydrophobic fungal conidia and the insect integument, thereby facilitating attachment, germination, and infection efficiency [28]. In addition to improving adhesion, oil-based formulations have been reported to enhance EPF performance by improving conidial distribution, facilitating cuticle penetration, and protecting fungal propagules from environmental stressors such as ultraviolet radiation and low humidity [29,30]. These properties collectively contribute to increased infectivity and persistence of fungal propagules under laboratory and field conditions. Previous studies have also indicated that certain oils may possess intrinsic insecticidal activity [31]. For instance, Abdel-Shafy and Soliman [32] reported that essential oils from *Lavandula officinalis*, *Mentha piperita*, and *Ocimum basilicum* caused significant mortality across multiple developmental stages of *Boophilus annulatus*. Similarly, Camargo et al. [33] demonstrated that oil-based formulations of *Metarhizium anisopliae* and *Beauveria bassiana* achieved high mortality against *Rhipicephalus microplus* larvae, while mortality observed in oil-only controls suggested a direct contribution of oil to insect mortality. Furthermore, Umaru and Simarani [34] reported lower LC_50_ values and faster LT_50_ values for oil-formulated EPF compared with aqueous formulations against *Elasmolomus pallens*. Their study also demonstrated enhanced activities of protease, chitinase, and lipase enzymes in oil-based formulations, suggesting that oils may indirectly improve fungal virulence by facilitating host penetration. Collectively, these findings indicate that oil-based formulations can substantially improve the insecticidal performance of EPF through enhanced adhesion, protection, and infection efficiency.

High efficacy against *P. xylostella* was observed in treatments combining EPF with pesticides, where complete mortality (100%) was achieved under laboratory conditions. Pesticide-alone treatments likewise resulted in complete mortality, indicating that both treatments were highly effective under laboratory conditions. Interestingly, observational assessments suggested that complete mortality was achieved earlier in the combined treatment, whereas LT_50_ analysis estimated a lower LT_50_ value for the pesticide-alone treatment. This difference highlights that treatment performance may vary depending on the metric used to assess mortality dynamics. Therefore, although both treatments ultimately achieved the same level of mortality, their patterns of mortality progression may differ. Similar improvements in efficacy following combined EPF–insecticide applications have been reported previously against several lepidopteran pests [35,36,37]. The different mortality responses observed between the combined treatment and pesticide-alone treatment may be attributable to the complementary modes of action of EPF and emamectin benzoate. Emamectin benzoate primarily targets glutamate-gated chloride channels and gamma-aminobutyric acid (GABA) receptors, resulting in paralysis and eventual insect death [38]. In addition, previous studies have suggested that emamectin benzoate may disrupt gut microbial communities, damage gut tissues, and impair antioxidant and immune-related functions in insects [39]. Such physiological disruptions may weaken host defense mechanisms and increase susceptibility to fungal infection. In contrast, EPF infect the host through direct cuticular penetration followed by proliferation within the hemocoel and production of toxic metabolites [40]. Therefore, the combined application may improve insect suppression by simultaneously affecting insect neurological function and reducing host resistance to fungal colonization. Similar interactions between *B. bassiana* and emamectin benzoate have been previously reported, where combined applications enhanced mortality and disrupted insect development compared with individual treatments [13]. These findings support the potential integration of EPF with reduced pesticide inputs within IPM programs, although additional studies are needed to determine whether these interactions are truly synergistic.

In greenhouse experiments, a similar mortality trend was observed, where combined oil-formulated EPF and pesticide treatments produced the highest mortality (approximately 70%) against third-instar larvae of *P. xylostella*. However, overall mortality under greenhouse conditions remained lower than that under laboratory conditions, highlighting the influence of environmental factors on fungal survival, germination, and infectivity under semi-natural conditions [21,41]. As the laboratory and greenhouse experiments were conducted under different environmental conditions, direct statistical comparisons between the two bioassays were not performed in the current study. Nevertheless, the reduced efficacy observed under greenhouse conditions highlights the influence of environmental variability on EPF performance and emphasizes the importance of optimizing formulations for semi-field and field applications. Reduced efficacy under greenhouse conditions may also be associated with uneven spray deposition, degradation of active compounds under light exposure, and reduced persistence on foliage surfaces.

Although the current study demonstrated encouraging biological activity of the nanoparticle-like fungal formulations against *P. xylostella*, several limitations should be acknowledged. In the present study, nanoparticle formation was inferred primarily from visual color changes during the biosynthesis process, whereas detailed physicochemical characterization, including transmission electron microscopy (TEM), scanning electron microscopy (SEM), dynamic light scattering (DLS), X-ray diffraction (XRD), and Fourier-transform infrared spectroscopy (FTIR), was not conducted because of facility limitations. Therefore, the synthesized materials should be considered preliminary nanoparticle preparations requiring further physicochemical characterization in future studies. Additionally, the current study primarily focused on evaluating the biocontrol efficacy of EPF-based formulations against *P. xylostella* and did not investigate the underlying mechanisms of nanoparticle–fungus interactions or fungal responses to pesticide exposure. Therefore, the mechanisms underlying the enhanced efficacy observed in the combined treatments remain unclear and warrant further investigation. Future studies involving transcriptomic, proteomic, and gene expression analyses may help elucidate the physiological and molecular interactions contributing to improved fungal pathogenicity and increased insect mortality.

## Figures and Tables

**Table 1:** Details of the experimental treatments used against the *Plutella xylostella.*

Treatment Code	Formulation Details	Concentrations
T1	Putative copper nanoparticles (CuNPs)	5, 10, and 15 µg/mL
T2	Putative zinc nanoparticles (ZnNPs)	5, 10, and 15 µg/mL
T3	Oil-emulsified EPF	1 × 10^6^, 1 × 10^7^, and 1 × 10^8^ conidia/mL
T4	Oil-emulsified EPF + Emamectin benzoate (2.15% EC; Sinon Corporation, Taipei, Taiwan)	1 × 10^6^ + 6, 1 × 10^7^ + 4, and 1 × 10^8^ + 2 (conidia/mL + ppm)
T5	Emamectin benzoate	2, 4, and 6 ppm
T6	Fresh EPF culture	1 × 10^6^, 1 × 10^7^, and 1 × 10^8^ conidia/mL
T7	Water control	_

Note: Putative copper nanoparticles (CuNPs) and zinc nanoparticles ZnNPs refer to fungal-mediated copper- and zinc-containing materials generated through the nanoparticle synthesis procedures described in Section 2.2.

**Table 2:** The LC_50_ values (conidia/mL or µg/mL) of the various treatments against third-instar larvae of *Plutella xylostella* were estimated using probit analysis.

Treatments	LC_50_	95% CI		Slope ± SE	χ^2^	*p*-Value
		Lower	Upper			
Putative copper nanoparticles (CuNPs)	9.004	6.606843	12.150371	0.931 ± 0.081	630.218	<0.001
Putative zinc nanoparticle (ZnNPs)	7.176696	5.899232	8.303586	1.671 ± 0.083	634.55	<0.002
Oil fungus	3.74 × 10^6^	1.66 × 10^6^	6.84 × 10^6^	0.404 ± 0.020	636.39	<0.002
Oil fungus + Pesticides	NE	NE	NE	NE	NE	NE
Pesticides	0.088	-	-	0.704 ± 0.102	2955.16	<0.001
Fungus	6.91 × 10^6^	2.62 × 10^6^	1.56 × 10^7^	0.411 ± 0.020	1166.23	<0.001

Note: NE: Not estimated. LC_50_ = lethal concentrations killing of 50% larvae. CI = 95% confidence interval. Oil fungus denotes oil-emulsified fungal formulation, and oil fungus + pesticides indicate the combined treatment.

**Table 3:** The LT_50_ (days) values for the various treatments against third-instar larvae of *Plutella xylostella* were estimated using probit analysis.

Treatments	LT_50_ (Estimate)	95% CI		Slope ± SE	χ^2^	*p*-Value
		Lower	Upper			
Putative copper nanoparticles (CuNPs)	5.6	2.99	9.04	0.973 ± 0.082	296.728	<0.001
Putative zinc nanoparticle (ZnNPs)	5.669	4.261	7.26	1.761 ± 0.084	250.335	<0.004
Oil fungus	6.08	5.55	6.52	0.426 ± 0.021	248.94	<0.001
Oil fungus + Pesticides	0.83	0.48	1.22	6.999 ± 0.209	34.968	0.984
Pesticides	0.18	0.04	0.39	1.434 ± 0.148	159.507	<0.001
Fungus	6.25	5.36	6.96	0.440 ± 0.021	706.57	<0.001

Note: CI = confidence interval, LT_50_: lethal time at which 50% larval mortality occurred. Oil fungus denotes oil-emulsified fungal formulation, and oil fungus + pesticides indicate the combined treatment.

## Data Availability

The original contributions presented in this study are included in the article. Further inquiries can be directed to the corresponding author.
